# A Scoping Review of the Conceptual Differentiation of Technology for Healthy Aging

**DOI:** 10.1093/geront/gnaa051

**Published:** 2021-07-29

**Authors:** Hansuk Kim, Sarah Kelly, Louise Lafortune, Carol Brayne

**Affiliations:** 1 Cambridge Institute of Public Health, School of Clinical Medicine, University of Cambridge, Cambridge, UK; 2 Ministry of Health and Welfare, Sejong, Republic of Korea; 3 THIS Institute, University of Cambridge, Cambridge, UK

**Keywords:** Concept, Framework, Public health, Strategy

## Abstract

**Background and Objectives:**

With the emergence of healthy aging as a key societal issue in recent decades, technology has often been proposed as a solution to the challenges faced by aging societies. From a public health perspective, however, aging-related technologies have been inconsistently conceptualized and ill-defined. By examining how relevant concepts in “technology for aging” have been developed to date, we hope to identify gaps and begin clarifying the topic.

**Research Design and Methods:**

We conducted a scoping review according to PRISMA-ScR, drawing on PubMed and Embase. We selected articles that directly reported concepts of technology for aging, or from which such concepts could be inferred.

**Results:**

We identified 43 articles, most of which were narrative reviews (*n* = 31). Concepts of technology for aging were presented in diverse ways with some overlap. Most studies provided some terminology (*n* = 36), but with little conceptual uniformity. Conceptual discourse was often focused on the aging agenda; while technological aspects were poorly defined. A conceptual framework from a public health perspective was derived from 8 articles—it showed that technology strategies do not take a population approach.

**Discussion and Implications:**

While the potential of “technology for aging” is vast, its real capacity to deliver a desirable life for older people remains underdeveloped. Clearer concepts and realistic goals at population level are lacking. Efficient investment must be made throughout the social system, and technology needs to be integrated via macro-level practices.

As global life expectancy at birth has risen from 47.0 years (1950–1955) to 70.8 years (2010–2015) (UN, 2017), developments in science and technology have increasingly promised to mitigate aging-related decline in quality of life (QoL) and rises in health and social care costs (Pilotto, Boi, & Petermans, 2018).

The potential of technology for addressing the challenges of aging populations has been studied since the 1980s (Peine & Neven, 2019). In the 2000s, when information and communication technology (ICT) experienced explosive growth, the topic increasingly attracted research interest from the many diverse academic disciplines that address aging. A wide range of major research themes emerged, from the perception, acceptance, and usability of technologies to the challenge of ensuring that older people are not excluded from technology-engaged living. Standards of evaluation and assessment, however, have not kept pace with rapid technological change (Schulz et al., 2015), in part because developers have placed greater emphasis on feasibility rather than effectiveness or formal evaluation (Satariano, Scharlach, & Lindeman, 2014). As a result, the effectiveness and value of technology for healthy aging has yet to be fully demonstrated (Blaschke, Freddolino, & Mullen, 2009; Satariano et al., 2014; Schulz et al., 2015).

Despite limited current evidence, there are grounds for optimism, given how successfully technology has infiltrated nearly all aspects of our lives (Nehmer, Lindenberger, & Steinhagen-Thiessen, 2010). The conceptualization of the nature and role of technology in aging, however, remains elusive (Pilotto et al., 2018; Schulz et al., 2015), with no overarching review to date. This lack of attention to concept development has made it difficult to provide an overall view of the technological landscape and a clear vision of what constitutes actual benefit and demand. Nor can it clearly be determined which technologies work for whom, or when and how they should be implemented (Petermans & Piau, 2017). The resulting absence of systematic prioritization across technologies makes it difficult to allocate resources according to individual-, community-, and/or societal-level benefit, and creates a vicious cycle that hinders our ability to demonstrate the effectiveness of technology for aging well. With a vast array of research disciplines and approaches involved, there is a need to define and differentiate the myriad ways technology can support individuals to age healthily, and allow aging communities to thrive.

The WHO framework on “active aging” (WHO, 2002) and the subsequent action-oriented report that framed the concept “healthy aging” (2015) took a public health perspective, moving away from the absence of diseases model of health. Both emphasized the importance of social, economic, and built environments as core determinants of healthy aging for individuals and whole communities. As a result, the pressing need to tackle the challenges of an aging society is often framed in public health terms. There has also been increased awareness of the fast-changing environment in which we live—highlighting emerging risks but also the potential of technology to fully realize “aging in place.” To date, the specifics of this exhortation have not been clearly defined, leaving lingering uncertainty about how technology should or could be integrated into the public health sector.

The public health perspective usually takes a broad approach and focuses on the entire spectrum of factors that may influence the health of whole or specific populations, including preventative health care, individual health behaviors, social, environmental, and economic factors, policies, and even genetic determinants of health. Taking a whole-systems public health approach allows us to map and understand the different pathways and most effective strategies by which technology for aging can be deployed for the benefit of whole communities and individuals within them. This contrasts with the bulk of scientific research conducted to date on technology and aging, which has generally focused on the individual perspective, that is, on the functional relationship between the technology and the older person, without fully considering the complex nature of how individuals interact with and within their environment.

This scoping review is the first to focus on the conceptual basis of technology for aging from a public health perspective. To help provide greater clarity, this review explores existing concepts of technology related to aging where definitions, terminology, and scope vary greatly. To capture the varied terminology that has previously been used, here we use “technology for aging” as a collective term for the entire field, including, but not limited to, such concepts as ICT, e-health, assistive technology, and smart technology when applied to aging and older people. The review also presents whether and how these concepts have considered the spectrum of factors that influence how whole or specific populations interact with or benefit from technology. Finally, it presents a conceptual framework that brings together current approaches that capture public health domains. This framework aims to provide a first step to understand and articulate the gap between current research on the role, effectiveness, and value of technology for aging and the realities of older people in lived life contexts. This is essential to inform what evidence needs to be developed to inform policy and practice for the benefit of whole populations.

## Research Design and Methods

Scoping reviews map the literature and identify gaps between knowledge and trends in a particular research area. This technology for aging review was conducted according to the classical stages (Arksey & O’Malley, 2005; Peters et al., 2015) and reported following PRISMA-ScR (Tricco et al., 2018) (Supplementary Table I).

### Identifying the Research Question

The lead author conducted preliminary searches on the relationship between aging and technology to refine the research question. Repeated searches were conducted for a wide range of technology terms combined with fulltext review of selected articles to scope how concepts or theories of technology for aging were presented. Many studies focused on acceptance and barriers after the explosive growth of ICT, with few referring to concepts or theories. Key terms varied, and some studies provided a conceptual approach alongside contextual background knowledge. Preliminary scoping searches also included specific terms such as “digital health” and “m-health.” Articles identified with these terms focused on the effectiveness, acceptance, and barriers to individual technologies with no examination of conceptual approaches to technology and aging. The researchers’ academic disciplines generally informed the way technology was described. Even when studies were not based on comprehensive research, the concepts of technology for aging could be examined.

We identified the following research questions:

(1) How is “technology for aging” defined by researchers?
(2) Using a public health perspective, can we establish a concept of technology for aging that promotes healthy aging, and create a public health-orientated conceptual framework?
(3) From the results of this scoping review, is it possible to derive public health implications for how technology development can contribute to an aging society?

## Identifying Relevant Studies

### Searching Electronic Databases

On February 8, 2019, we conducted searches in two major health-related databases: PubMed and Embase. A leading and clear concept of “technology for aging” could not be identified by the preliminary searches, so comprehensive searches were conducted using “technolog*” in combination with broad age- and concept-related search terms or “public health” (Table 1). Additionally, we searched for titles or abstracts containing the term “gerontechnology,” regardless of other keywords. No limitation was set on the publication year. As a result, we retrieved 1,774 articles, with a further 53 articles from the preliminary searches and other sources (e.g., Google Scholar). Only English-language articles were selected. Letters, commentaries, theses, and books were excluded.

**Table 1.** Search Terms

1) Population (title)	2) Intervention/exposure (title/abstract)	3) Outcome (all fields)	4) Environment (all fields)
Aging/ aging OR Older OR “old age” OR Senior 1) AND 2) AND 3) PLUS 1) AND 2) AND 4)	Technolog*	Concept* OR Definition OR Framework OR Model OR Theor* OR Classif*	“Public health”

*Note:* *Denotes wildcard symbol.

### Screening

The inclusion and exclusion criteria applied during the three screening stages (title, abstract, and full text) are shown in Table 2. Instead of setting narrow inclusion criteria from the start, we applied exclusion criteria in a stepwise manner to select as many relevant studies as possible. This approach was designed to identify the concepts in the literature without unnecessarily broadening the scope.

**Table 2.** Inclusion and Exclusion Criteria

Inclusion criteria	Exclusion criteria
1.All types of studies and reviews 2.The subject of the study was the older population 3.Application of technology within a public health setting 4.The concept of technology for aging is clearly discussed in the text (concept and/or definition), or it could be surmised through the context (framework, model, theory, and/or classification)	1.Letters, commentaries, theses, and books 2.Laboratory technology or techniques 3.Older population in unique environment 4.Studies that compared differences (i.e., in effectiveness) within older populations and between older population and other groups 5.Technology referring to measuring equipment of intervention outcomes 6.Technology applied in clinical settings and technology used to solve a medical problem 7.Technology acceptance and barriers being the main context 8.Effectiveness-focused studies without conceptual approach

At the title screening stage, studies on older populations in unique environments (e.g., older migrants) or those assessing the effectiveness of a particular technology within older populations (e.g., those with or without dementia or cognitive decline) and between older populations and other groups (such as younger people) were excluded as they generally did not refer to or have a clearly stated conceptual basis. We also excluded studies where the technologies were not directly relevant to the scope of this review, such as laboratory technology, equipment for outcome measurement of interventions (e.g., imaging devices), and electronic health records for monitoring.

Abstract screening focused on setting and context. Studies in hospital settings and those that implemented technology to solve medical problems were excluded. Articles on technology acceptance and its barriers were also excluded, as these would be outside our focus on the conceptual relationship between aging and technology. However, because the environment and purpose of technology for aging can vary, we found some articles difficult to exclude solely on the abstract. Such articles were reviewed again in full-text screening.

In total, 227 articles were included for full-text screening. Of these, 10 conference proceedings and six original articles were excluded because we could not locate a full text. Full-text screening was performed on 211 articles to determine whether they defined or described any concepts of technology for aging. Articles were included if the conceptual perspective of the authors could be determined, even if there was no direct mention of concepts. 

### Selecting Studies and Charting the Data

**Figure 1.**
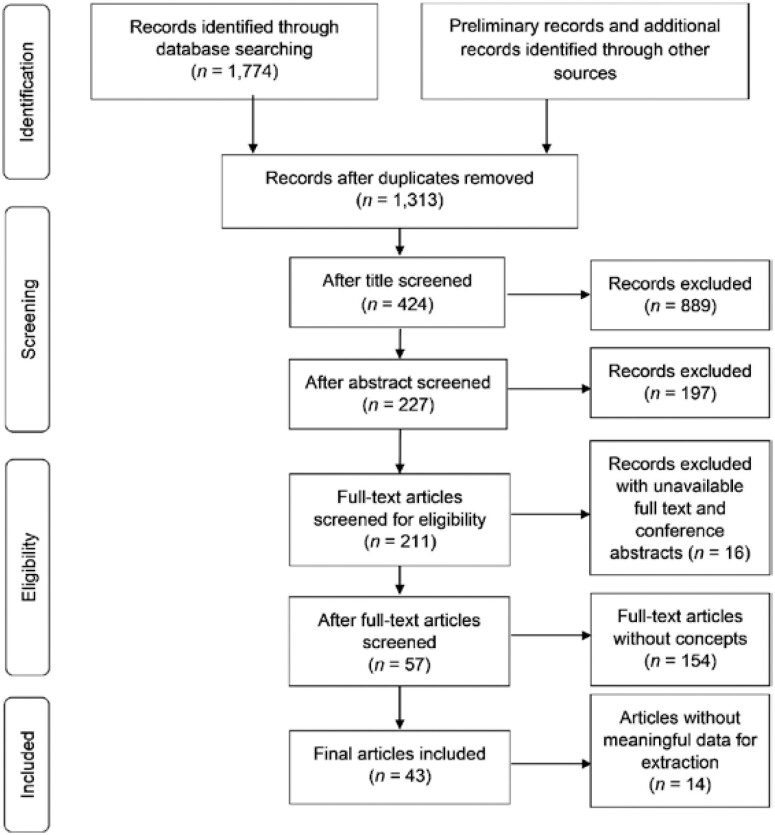
Flow diagram for the scoping review process.

Fifty-seven studies were initially identified from the screening for data extraction. The most decisive factor for study selection was whether the author clearly presented the concept, framework, theory, model, and/or classification of technology for aging. During the data extraction stage, a further 14 articles did not have meaningful data for extraction. Therefore, data were extracted from 43 articles that passed through these stages, as illustrated in Figure 1. The following variables were extracted: (a) authors; (b) title; (c) year of publication; (d) country of origin; (e) authors’ subject; (f) purpose; (g) methods; (h) technology type; (i) key findings; (j) concept of technology for aging; (k) presentation type of concepts; and (l) implications for public health. Data were extracted into Microsoft Excel.

## Results

Of the 43 included articles, 37 were published after 2005. Twenty-one articles had first authors from European countries, with a further 16 from the United States. There were 14 interdisciplinary studies with cooperation between two or more academic disciplines, at least one of which was a technology-related discipline (e.g., engineering, computer science). Thirty-one articles were narrative or descriptive reviews. The concepts of technology for aging were presented in diverse ways, including (a) early theory development (*n* = 3); (b) using specific terminology (*n* = 36); (c) using a classification matrix (*n* = 4); (d) presenting a technology hierarchy (*n* = 4); and (e) others (*n* = 3). A public health perspective was inferred from eight articles. Articles that presented the concept in multiple ways were counted in duplicate (Table 3).

**Table 3.** Descriptive Characteristics of the Included Articles (*n* = 43)

Authors	Title	Country of origin	Interdisciplinary approach^a^	Presentation type of concept
*Narrative review (n = 31)*				
Benefield and Holzclaw (2014) Bouma (1998)	Aging in place: merging desire with reality	United States	Noninterdisciplinary	Terminology: aging in place
Bouma et al. (2007)	Gerontechnology: emerging technologies and their impact on aging in society	The Netherlands	Interdisciplinary	Terminology: gerontechnology
	Gerontechnology in perspective	The Netherlands, Finland, United States	Noninterdisciplinary	Terminology: gerontechnology Classification matrix
Cabrita et al. (2018)	Persuasive technology to support active and healthy aging: an exploration of past, present, and future	The Netherlands	Interdisciplinary	Public health perspective
Cheek et al. (2005)	Aging well with smart technology	United States	Noninterdisciplinary	Terminology: smart technology
Fozard (1996)	Aging and technology: a developmental view	United States	Noninterdisciplinary	Early theory Terminology: gerontechnology
Fozard and Heikkinen (1998)	Maintaining movement ability in old age: challenges for gerontechnology	United States, Finland	Noninterdisciplinary	Terminology: gerontechnology
Gandarillas and Goswami (2018)	Merging current health care trends: innovative perspective in aging care	Spain, Austria, Slovenia	Noninterdisciplinary	Terminology: smart technology (e-health) Public health perspective
Garçon et al. (2016)	Medical and assistive health technology: meeting the needs of the aging populations	Switzerland, Australia, Sweden	Noninterdisciplinary	Terminology: assistive technology
Haber (1986)	Technology in aging	United States	Noninterdisciplinary	Early theory Technology hierarchy
Hanson et al. (2013)	Emerging technologies to support independent living of older adults at risk	United States	Noninterdisciplinary	Terminology: smart technology (home telecare or telehealth)
Harte et al. (2014)	Human-centered design considerations for connected health devices for the older adult	Ireland, Spain, United States	Interdisciplinary	Terminology: smart technology (connected health)
Joyce and Loe (2010)	A sociological approach to aging, technology, and health	United States	Noninterdisciplinary	Sociological approach
Knight et al. (2018)	Systems to harness digital footprint to elucidate and facilitate aging in place	Australia	Noninterdisciplinary	Terminology: aging in place
Koch (2010)	Healthy aging supported by technology—a cross- disciplinary research challenge	Sweden	Noninterdisciplinary	Terminology: AAL technology Public health perspective
Layzell et al. (2009)	The elderly demographic time bomb—sharing the load with the actively aging: can e-health technologies help defuse it	United Kingdom	Interdisciplinary	Terminology: smart technology (e-health, smart home, smart environment)
Lindenberger et al. (2008)	Psychological principles of successful aging technologies: a mini-review	Germany	Interdisciplinary	Terminology: intelligent assistive technology (psychological principle)
Majumder et al. (2017)	Smart homes for elderly health care—recent advances and research challenges	Canada, Iran, Sweden	Interdisciplinary	Terminology: smart home technology
Micera et al. (2008)	Gerontechnology	Italy, United States, Japan	Noninterdisciplinary	Terminology: gerontechnology Technology hierarchy
Mortenson et al. (2015)	The power(s) of observation: theoretical perspectives on surveillance technologies and older people	Canada	Noninterdisciplinary	Terminology: AAL technology (surveillance technology)
Peine and Neven (2019)	From intervention to co-constitution: new directions in theorizing about aging and technology	The Netherlands	Noninterdisciplinary	Sociogerontological approach
Piau et al. (2014)	Aging society and gerontechnology: a solution for an independent living	France	Noninterdisciplinary	Terminology: gerontechnology
Rashidi and Mihailidis (2013)	A survey on ambient-assisted living tools for older adults	United States, Canada	Noninterdisciplinary	Terminology: AAL technology
Riva et al. (2014)	Positive technology for healthy living and active aging	Italy	Interdisciplinary	Terminology: positive technology Public health perspective
Rodeschini (2011)	Gerontechnology: a new kind of care for aging? An analysis of the relationship between older people and technology	Italy	Noninterdisciplinary	Terminology: gerontechnology
Rogers and Fisk (2010)	Toward a psychological science of advanced technology design for older adults	United States	Noninterdisciplinary	Terminology: aging in place (ambient technology)
Satariano et al. (2014)	Aging, place, and technology: toward improving access and wellness in older populations	United States	Noninterdisciplinary	Terminology: aging in place Classification matrix Public health perspective
Schulz et al. (2015)	Advancing the aging and technology agenda in gerontology	United States	Noninterdisciplinary	Terminology: QoL technology Classification matrix
Smith (1990)	Human factors and aging: an overview of research needs and application opportunities	United States	Noninterdisciplinary	Early theory
Tuchetti et al. (2011)	Technology and innovative services	Italy	Interdisciplinary	Technology hierarchy Public health perspective
van Bronswijk et al. (2009)	Defining gerontechnology for R&D purposes	The Netherlands, United States	Interdisciplinary	Terminology: gerontechnology Classification matrix Public health perspective
*Qualitative study (n = 7)*				
Bailey and Sheehan (2009)	Technology, older persons’ perspectives and the anthropological ethnographic lens	Ireland	Noninterdisciplinary	Anthropological approach
Chen and Chan (2013)	Use or nonuse of gerontechnology—a qualitative study	Hong Kong	Noninterdisciplinary	Terminology: gerontechnology
Courtney et al. (2008)	Needing smart home technologies: the perspectives of older adults in continuing care retirement communities	United States	Interdisciplinary	Terminology: smart home technology
Kelly et al. (2014)	Responding to home maintenance challenge scenarios: the role of selection, optimization, and compensation in aging in place	United States	Noninterdisciplinary	Terminology: aging in place
Peek et al. (2016)	Older adults’ reasons for using technology while aging in place	The Netherlands	Interdisciplinary	Terminology: aging in place
Peek et al. (2016)	What it takes to successfully implement technology for aging in place: focus groups with stakeholders	The Netherlands, Singapore	Interdisciplinary	Terminology: aging in place
Pekkarinen et al. (2013)	Review: roles and functions of user-oriented gerontechnology: mStick and hStick	Finland	Interdisciplinary	Terminology: gerontechnology
*Scoping/systematic review (n = 4)*				
Blackman et al. (2016)	Ambient-assisted living technologies for aging well: a scoping review	Canada, Sweden, United Kingdom	Interdisciplinary	Terminology: AAL technology Technology hierarchy
Demiris and Hensel (2008)	Technologies for an aging society: a systematic review of “smart home” applications	United States	Noninterdisciplinary	Terminology: smart technology (smart home)
Kim and Lee (2017)	Smart devices for older adults managing chronic disease: a scoping review	Canada	Noninterdisciplinary	Terminology: smart technology (m-health)
Reeder et al. (2013)	Framing the evidence for health smart homes and home-based consumer health technologies as a public health intervention for independent aging: a systematic review *nterventional study (n = 1)*	United States	Noninterdisciplinary	Terminology: smart home technology Public health perspective
*Mixed: qualitative and i*				
Dahler (2018)	Welfare technologies and aging bodies: various ways of practicing autonomy	Denmark	Noninterdisciplinary	Terminology: welfare technology

*Note:* AAL = ambient-assisted living; QoL = quality of life.

^a^Included at least one technology-related discipline (e.g., engineering, computer science) from authors’ institutions.

### Early Theory Development

Three articles described early theory on technology for aging. Early theories incorporated human factor analysis, ergonomic theory, and ecological approaches. Human factor analysis places importance on the mutual process of humans accepting machines, and recognizes technology as a factor affecting the living environments of older populations (Smith, 1990). Ergonomic theory emphasizes how the process of maintaining or restoring physical functions can be integrated with technology (Fozard, 1996). According to Haber (1986), the ecological approach focuses on the use of technology for improving the daily lives of older people through its application to activities, transportation and mobility, communication, workplace design, and recreation. It suggests that such an early approach can also be used to design the environments of older populations.

### Use of Terminology

**Figure 2.**
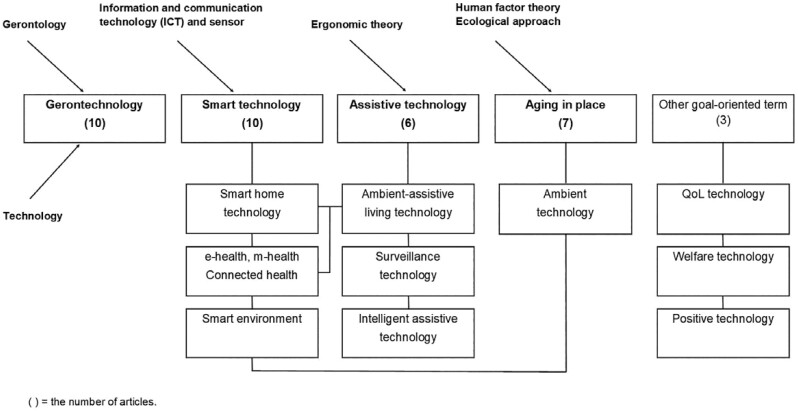
Specific terminology in the included articles.

A wide variety of terms have been used in the included research (*n* = 36), with a diversity and conceptual overlap that can cause confusion, namely because terms refer to specific technologies as well as the goal pursued by these technologies. Figure 2 presents how these have been grouped in four broad categories to illustrate the hugely diverse descriptions and concepts that have been used to describe technology for aging: gerontechnology, smart technology, assistive technology, and technologies to support “aging in place.”

### Gerontechnology

Ten articles dealt with this term, coined by Graafmans and Brouwers in 1989 (as cited in Fozard, 1996). Its dual etymology implies the interaction of two very different developmental processes: societal aging and technological advancement (Bouma, Fozard, Bouwhuis, & Taipale, 2007). At the same time, gerontechnology is defined as technology that meets the demands and interests of older people, thereby allowing them to adapt to their environments by preserving function or delaying the loss of capacity (Fozard & Heikkinen, 1998). Accordingly, the concept of gerontechnology can include activities such as education, training, and counseling to meet social and psychological demand (Fozard, 1996). van Bronswijk et al. (2009) stipulated that gerontechnology targets successful aging by ensuring technology responds to the demands of older populations and promotes quality of life (QoL). In practice, gerontechnology often encourages social participation and independent living among older people through electronic or digital products or services (Chen & Chan, 2013; Pekkarinen et al., 2013). Some articles placed significance on the representativeness of the term “gerontechnology” within related research (Micera, Bonato, & Tamura, 2008; Piau, Campo, Rumeau, Vellas, & Nourhashemi, 2014; Rodeschini, 2011). While the described applications of gerontechnology can include housing, communication, transportation, health, and work (Bouma, 1998), the scope and definition in relation to public health is unclear and several researchers have concluded that the concept of gerontechnology is still theoretically immature (Blackman et al., 2016; Schulz et al., 2015).

### Smart Technologies

As defined in 10 articles, this typically refers to digital products and services aimed at achieving “aging in place,” though the term is also used in conjunction with the concept of “assistive technology.” These technologies establish an environment in which devices are interconnected with home automation and may allow communication to the outside world from home (Cheek, Nikpour, & Nowlin, 2005; Courtney, Demiris, Ranz, & Skubic, 2008). At the core of smart home technology are sensors that support activities of daily living by monitoring movements within the home (Hanson, Takahashi, & Pecina, 2013). “Health-related smart home” refers to technology that is integrated into the living space, while “home-based consumer health technology” describes devices that are not integrated (Reeder et al., 2013). Meanwhile, concepts such as “e-health” and “m-health” are applied to data collection and sharing through digital devices for early detection and disease management (Gandarillas & Goswami, 2018; Kim & Lee, 2017). “Smart home” is a comprehensive concept that refers to a complete system aimed at achieving health, safety, and well-being, usually by enabling remote surveillance of health status (Demiris & Hensel, 2008; Majumder et al., 2017). “Connected health” includes a concept encompassing all terminology associated with smart technology, including e-health, telehealth, telemedicine, smart home technology, digital health, and remote care (Harte et al., 2014). The wider concept of “smart environments” has been proposed because of concern about the possibility of individuals being kept at home due to dependence on technology. It includes community activities, such as public transport and shopping (Layzell, Manning, & Benton, 2009).

### Assistive Technology

One of the most commonly used umbrella terms in technology for aging, this was defined in six articles. Assistive technology is grounded in ergonomic theory. The most well-known definition is technology realized through assistive devices or services that improve the physical function and independence of individuals (Garçon et al., 2016). Low-technology level devices, such as wheelchairs and walkers, are included in this definition, but are not considered specific to technology for aging because they can also be used for individuals with disabilities. “Ambientassisted living (AAL) technology” is a higher-level term that overcomes this limitation. AAL is a concept with the goal of enhancing efficiency and productivity through extending the time older people spend in familiar environments (Koch, 2010). AAL technology is used for lifestyle management, early detection of diseases, assistance in daily life, and disease management. Digital technology, smart homes, assistive robotics, e-textiles, and wearable sensors can all be considered AAL technologies, making it a broad term (Rashidi & Mihailidis, 2013). Therefore, AAL technology is a goal-oriented term beyond low-level assistive technology, encompassing “third-generation technology” that supports the daily lives of individuals through ambient systems (Blackman et al., 2016). It can also be considered a “surveillance technology” that monitors within familiar environments (Mortenson, Sixsmith, & Woolrych, 2015). Meanwhile, “intelligent assistive technology” is a complex concept that allows older people to recognize aging in terms of psychological principles. It strikes a balance between environmental support and individual motivation, and is a life-span technology that assists people from their middle to later years (Lindenberger, Lövdén, Schellenbach, Li, & Krüger, 2008).

### Aging in Place

Seven articles focused on this goal-oriented term. Though it does not itself contain a technology domain, it is commonly associated with—and achieved by—smart or assistive technologies. Aging in place enhances the interaction between older people and their environment, with technology taking an important role in helping them remain independently active and healthy (Benefield & Holzclaw, 2014; Peek, Luijkx, et al., 2016; Peek, Wouters, Luijkx, & Vrijhoef, 2016). Older users expect to be able to resolve declines in physical function through technology (Kelly, Fausset, Rogers, & Fisk, 2014). Technology is also an important mechanism for filling the gap between the knowledge and the reality of aging by compensating for environmental and behavioral changes, and thus facilitating aging in place (Knight et al., 2018; Satariano et al., 2014). “Ambient technology”—which drops the word “assistive” from AAL technology—is indeed closer to smart technology than to assistive technology. It refers to higher-level technology that interacts with the environment, whereas assistive technology refers to tools used by individuals (Rogers & Fisk, 2010). Another term in this article, “ambient intelligence,” describes often intangible technologies imbedded in “smart” reactive environments.

Some authors introduced less widely used terms to explain that technology for aging has been influential to an aging society. Schulz et al. (2015) stated that technology for aging belongs wholly to “QoL technology” because all human functions develop and change across the life course, and technology is important for both older individuals and an aging society. Scandinavian countries have coined the concept of “welfare technology” as an expanded form of assistive technology. It can be a part of the welfare service being provided directly to older population, including innovative actions for addressing this demographic challenge (Dahler, 2018). Similarly, “positive technology” is a term used to support technology for strengthening the sustenance and restoration of individuals, organizations, and society. Although this concept originates from individual demand and experience, the social effect can be greater, depending on the recognition given to the positive influence of technology (Riva et al., 2014).

### Use of Classification Matrices

**Figure 3.**
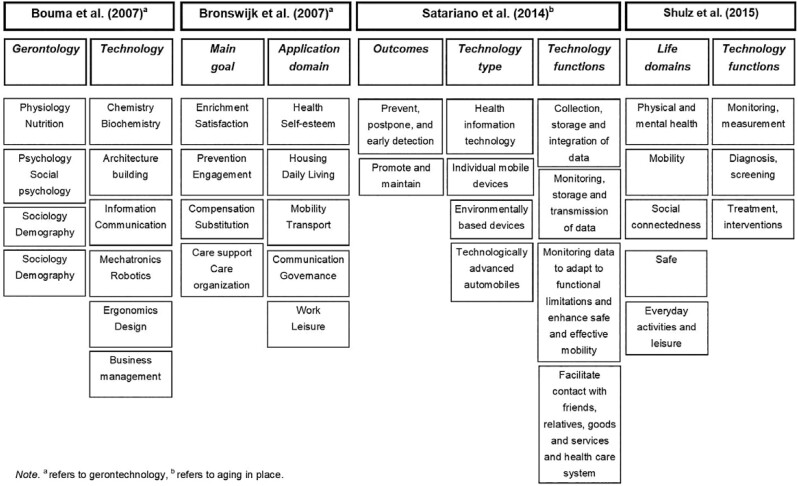
Domain classification matrices.

Four articles supplemented concepts with classification criteria of technology for aging (Figure 3). Classification matrices can differentiate the features of aging and technology and effectively show their relationships, allowing researchers to derive future research topics using a pragmatic approach beyond mere conceptual discourse.

First, Bouma et al. (2007) suggested a cross-fertilization matrix to explain the scope of gerontechnology. The classification categories consisted of the disciplines of gerontology and technology. A wide variety of sectors, from preventive nutrition to targeted marketing, were included within the scope of gerontechnology. One innovation of this matrix was that business management was actively pulled into the technology category.

van Bronswijk et al. (2009) formulated three gerontechnology matrices by modifying previous matrices, including the cross-fertilization matrix. One of these was based on an engineering perspective that classified according to the main goals of gerontechnology and various application domains. However, the application domains were still goal-oriented, such as health and self-esteem, mobility, and transport. For this reason, a subsequent study modified the matrix by highlighting the characteristics of technology (Peek, Wouters, et al., 2016).

Satariano et al. (2014) presented a classification matrix with three prominent technology categories: type of technology (e.g., information technology); outcomes expected through technology (e.g., prevention of falls); and functions of technology (e.g., monitoring of data).

Schulz et al. (2015) also presented a matrix using the technology function and key life domains.

Unfortunately, even when aspects of the technology are addressed they tend to be undifferentiated and overshadowed by goal orientation.

### Technology Hierarchy

Four articles presented hierarchies that can be helpful for understanding the actual type of technology for aging. Haber (1986) classified technology for aging into three levels: the lowest consists of objects modified for the convenience of older people, at mid-level are technologies that use energy and require simple manipulations, while high-level technologies were defined as new devices that are used in high-skill health care settings. Blackman et al. (2016) cited Doughty and Sixsmith in presenting a hierarchy of AAL technology, stating that first-generation AAL is technology such as wearable devices and emergency alarms, second-generation refers to home sensor technology and automated alarm systems, while third-generation allows for less obtrusive monitoring and support by integrating home sensors and wearable devices.

A more comprehensive technology classification was presented by Micera et al. (2008), in which the lowest grade referred to basic digital technology that assists connection and communication; the middle grade comprised software technology that expands knowledge, wearable devices, and smart home technology; bio robot technology was placed between the middle and highest grade, which consisted of systems that assist physical and cognitive autonomy such as eGovernance and eCommerce, remote health care, and AAL technology.

From this technology hierarchy, Turchetti, Micera, Cavallo, Odetti, and Dario (2011) propose third-generation eCare services that simulate how technology for aging can contribute to long-term care. This “third age and health care technology” consists of sensor technologies and remote monitoring ubiquitous robots that can help older users—even individuals with cognitive dysfunction—maintain daily life patterns.

### Public Health Perspectives

**Figure 4.**
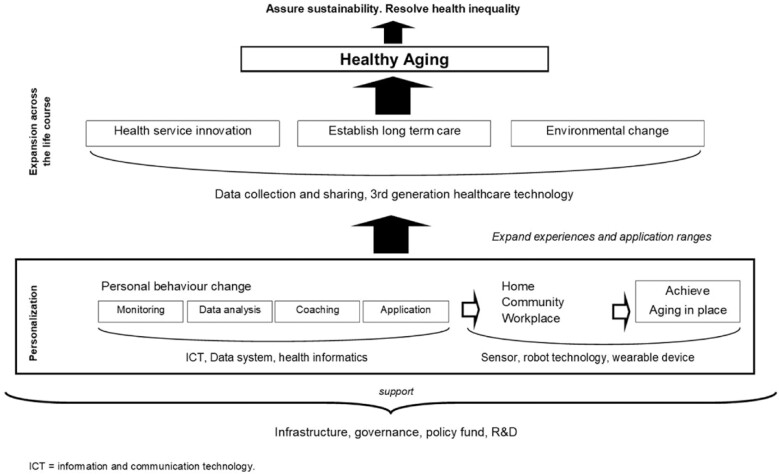
Conceptual framework of technology for healthy aging.

Among the 43 included articles, not one considered the spectrum of factors that influence how whole or specific populations interact with or benefit from technology. Public health was discussed only sporadically in relation to the goals that the technologies aimed to realize, the communities in which they were applied, and policy implications. Referencing the WHO framework (WHO, 2015), eight mentioned the role of technology in achieving challenging goals related to public health policy and implementation (Supplementary Table II). Building on the information presented in these articles, it is possible to develop an initial conceptual framework of the relationship between technology and healthy aging (Figure 4). The frame of user– technology–environment can be a useful starting point for a population-based perspective, because the technology can allow population-level interventions to be tailored to individual experience. Technology can thus be employed to change behavior *and* environment, specifically to meet the needs of each individual in specific contexts. To accomplish this, the diversity of older population’s needs and wants must be accurately considered through appropriate engagement, measurement, monitoring, and surveys that can establish who would benefit from which technology. ICT-based health informatics is important in this regard (Cabrita, Op den Akker, Tabak, Hermens, & Vollenbroek-Hutten, 2018; Gandarillas & Goswami, 2018). Such technology can be very complicated to operate at the population level because individuals, consumers and patients, service providers, technology developers, and policymakers all significantly influence how such technologies can provide benefits (while not doing harm).

It is also important to consider the health and social systems’ context in which the technology is implemented. Some studies have suggested that if an ICT-based individual databank could be established for sharing health information, this would lead to tailored services that meet the needs of individual older people (Cabrita et al., 2018; Gandarillas & Goswami, 2018). Once the potential benefit of an individual technology is mapped to the demand of older people, its implementation can be designed to help them maintain their lifestyles. Sensor technology and wearable devices, which are aimed at supporting older people’s social connectedness, could thus help sustain aging in place (Koch, 2010; Reeder et al., 2013; Riva et al., 2014; Satariano et al., 2014).

As noted above, an individual’s environment and conditions influence the personalization of technology; thus, aging in place is accompanied by environmental change. This might be in the expansion of the range of available technological strategies and tools, and the resulting expansion of the range of effects they have on personal experiences (Riva et al., 2014), in the context where stable resources for such a process to take place at the community level are secured (Reeder et al., 2013). The effectiveness of technology for aging can only be assured when this approach is applied across the entire life course (i.e., current and future older generations; van Bronswijk et al., 2009). Technologies that contribute to such an expansion include ICT-based data collection and sharing, as well as third-generation health care technology (Turchetti et al., 2011). The scaling of technology from the individual to the population level will be most beneficial if accompanied by quality standards, operational governance, public resources, legal and ethical regulations, promotional policies, and a vibrant, creative research sector. Moreover, the required support infrastructure will only be achieved through a multidisciplinary approach (Koch, 2010; Satariano et al., 2014; Turchetti et al., 2011; van Bronswijk et al., 2009).

If implemented with care, technology for aging can support health service innovation and changes in long-term care that optimize aging from full health to end of life. When a sufficient number of older people receive these benefits, a sustainable future can be assured, and health inequalities addressed (van Bronswijk et al., 2009), to some extent.

Three articles do not belong to any of the above categories. Bailey and Sheehan (2009), using an anthropological approach, found that user awareness of technology can improve QoL, well-being, and aging in place, and that technology can also be a vital part of social relationships. Joyce and Loe (2010) argued that “combining science and technology studies and sociology of health and illness frameworks together provides an empirical basis from which to analyze technogenarians in action—creatively negotiating technology and science to maintain independence and health across the lifecourse.” More recently, Peine and Neven (2019) advocated moving beyond an interventionist approach, in which aging is a target for technological interventions. They argue instead for broader research around aging and technology such as study of “how aging is already co-constituted by gerontechnology design, the socio-material practices it enacts, and the policy discourse around them.”

## Discussion and Implications

### How “Technology of Aging” Is Defined by Researchers

The concept of technology for aging has been presented in many diverse ways. Although a variety of terms has been used, findings indicate a disconnect between the understanding of aging as both person-level and population-level processes, and how technology can effectively be deployed to mitigate risks and capitalize on opportunities at both levels. This disconnect is in part due to high-level goaloriented concepts overlooking the specifics of populationlevel technologies and what is needed to implement them successfully. The concepts were consistently presented as supporting a desirable life for older people, but far less consistent about how to achieve this goal. These findings echo earlier reviews, which concluded that research in the field is often unclear (Schulz et al., 2015), pointing to the need for more robust conceptualization.

There are many reasons why it can be difficult to influence the world of technology through academic research. Technology is historically seen as a toolbox, and its usability is often addressed in exclusively pragmatic terms. However, rapid technological change has unpredictable societal effects and the implications of any particular technology can only be found by studying its widespread use and impact on human lifestyles. Moreover, individual technologies naturally fuse and overlap, making conceptual differentiation difficult. Technology itself is often categorized by the diverse functions it targets—cognitive, motor, and emotional—and it is challenging to adapt these classifications to include the specific needs of older people and the contexts they live in (Petermans & Piau, 2017). Moreover, the market dynamics that drive the technology sector are often at odds with the pace and nature of academia, even when a technology derives from academic advances.

From an economic perspective, there are two divergent views regarding older populations: one is that older populations’ huge purchasing power should be targeted, the other is that doing so can limit development by neglecting the scale and structure of the market as a whole. In technologically advanced countries, the older population has demonstrated sufficient purchasing power, critical views of products, and a range of interests (Bouma et al., 2007), yet there can be consumer resistance to targeting of the older population due to stigma about aging and its associated impairments (Petermans & Piau, 2017; Pilotto et al., 2018). In any case, targeting technologies at an older population may be more of a social priority than a market opportunity. It might be more fruitful to propose technology for “an aging society” as opposed to “the older population.” Technology strategies aimed at the entire life course, or goals for reducing impairments and improving function regardless of age, might be preferable for marketing and societal gains.

### Conceptual Framework From a Public Health Perspective

The development of technology to respond to an aging society is not a new topic on the policy agenda. In two Global Forums (WHO, 2013, 2016) that discussed how innovation can change communities and systems, the WHO has noted that countries worldwide are investing in technology to mitigate some of the consequences of aging. Such high-level policy statements can be useful to mobilize actors, but more in-depth thinking is needed to capture known heterogeneity of older populations and their needs and desires for technologies. It is also the case that any population’s potential to benefit from technology will vary enormously with differences in aging, pace of change, and economic status. The technology component of WHO’s “aging in place” strategy (WHO, 2013, 2016) aims to achieve aging in place through enhanced participation in design and better accessibility. However, the scope of technology for aging goes well beyond aging in place, and the true implementation of this approach requires consideration of far more than these factors, including improving applicability and accessibility of “proven” technologies (WHO, 2016).

In the literature we identified, technology is often framed as a “person-centered” concept, and the resulting diversity of frames is one key reason why a public health approach is critical. Among the 43 included articles, few articles specifically focused on the role of “technology for aging” for whole communities. Rather, research as mainly focused on individually targeted technology. Research on the relationship between aging and technology in public health research is rare, and studies that focus on the conceptualization of that relationship equally so. Still, using a broad and stepped approach in searching the literature, we identified eight articles that capture important dimensions relevant to public health. The initial conceptual framework we developed on the basis of these studies (Figure 4) exposes the gap between the current body of conceptual research, and how the policy goals are framed on one hand and technology developed on the other. It highlights that a clear technology strategy is missing. Furthermore, it shows how the gerontology-focused academic literature on technology for aging is undeveloped (Peine & Neven, 2019), explaining in part why it does not appear to have influenced public health actions and policies in the “real” world.

### Technology Strategy at the National Level

Technology will continue to advance and those entering older age will have a different attitude and experience from earlier generations (Rogers & Fisk, 2010). With the arrival of the Fourth Industrial Revolution, technology has accelerated its influence on the health and wealth of older people. Technology is still used as a tool, but is expanding into new realms including financial services, specifically internet banking (World Economic Forum, 2016). Lack of an appropriate technology strategy raises the question of whether policy is keeping up with fragmented developments of unknown value. Efficient investment must take place throughout the entire social system, and technology needs to be integrated with macro-level social work practices (Blaschke et al., 2009), as well as with innovations in health services, long-term care, and the environment (WHO, 2015).

Nations and regions need to tackle this challenge. The UK government unveiled grand plans to address future society through its Industrial Strategy (UK Government, 2017). One of these is the implementation of active innovations to meet the demands of an aging society. This strategy shows how individual emerging technologies (e.g., artificial intelligence and autonomous vehicles) are being funded and developed. However, action plans far beyond the R&D investments reported within the strategy are already overdue. Technology strategy is not just about which products should be developed for older people, it is also about what assists them to live well while maintaining their independence, and what can reduce social costs by replacing care resources. The full potential of technology for aging has not yet been adequately discussed or identified. If the older population is considered an active social resource, a technology strategy must be discussed in depth alongside specific social agendas, such as how technology might defer retirement.

### Implications for Public Health Action

Because public health action involves various public and private sectors, a clear conceptual differentiation of technology for aging requires considering the role of each sector. Research conducted to date does not meet that requirement. This adds to the long-standing concern in public health that continued advances in technology aimed at individuals—as opposed to whole communities—will lead to even greater inequality. As the public health response to inequality has often proved ineffective to date, a technology strategy requires a robust public health focus from the outset.

It is still necessary to establish evidence for the effectiveness of technology for aging. Given the interdisciplinary nature of this field, it will be difficult to establish methodological standards for technology evaluation. There is a need to set more realistic research goals at the population level and to continuously promote technology-based interventions with data collection and sharing (Beard & Bloom, 2015). Moreover, practical research involving health and social systems must be conducted to initiate innovations (WHO, 2016).

This scoping review presents the gaps and limitations of current research on the concept of “technology for aging.” It shows how concepts of technology for aging are used interchangeably in the scientific literature, with a lack of specificity. Moreover, it uniquely discusses the implications of this finding from a public health perspective. A scoping review attempts to capture the breadth of the research rather than aim for exhaustivity. As such, a limitation of this study is that relevant research will have been missed due to the way we conducted the searches, but also due to the lack of reference to any concept or theory in the title, abstract, and/or key words.

With this limit in mind, we presented a conceptual framework that highlights the current state of conceptualization applied to technology for aging in the scientific literature. Further work is recommended to establish a forward-looking conceptual framework, one that can guide research and action on the basis of well-characterized individual and community needs and expectation. As this evidence accrues, it will further inform the framework and offer a way of identifying gaps in evidence to inform policy and practice, which in turn helps identify research, evaluation, and implementation priorities. These are the key steps on the pathway to affect from technological innovation to the promotion of healthy aging at the population level.

In conclusion, this scoping review revealed that the concept of “technology for aging” has been used in a variety of ways. Conceptual uniformity is lacking, with much overlap between similar terminology and classification domains. Conceptual discourse tends to be goal-oriented, while actual technology domains are not well-differentiated. The conceptual framework synthesized from the articles showed that a technology strategy would go a long way in identifying what, when, and for whom technology of aging would benefit older populations the most. The value of technology for an aging society should be explored by working with various public health sectors to rethink, at the macro level, the place of technology in health and social care services. Through studies on the identification of demand and the development of technology that can be scaled to population level—plus the use of technology in the right place, at the right time through effective financial support—technology should be able to make sustained, positive contributions to health with aging.

## Supplementary Material

gnaa051_suppl_Supplementary_Materials
