# Supplementary material for: A Scoping Review of the Conceptual Differentiation of Technology for Healthy Aging
Source: Gerontologist. 2021 Jul 29;61(7):e345–69. doi: 10.1093/geront/gnaa051 (PMC8437509; doi:10.1093/geront/gnaa051)
Supplement: gnaa051_suppl_Supplementary_Materials [file gnaa051_suppl_supplementary_materials.docx]

Supplementary Materials

Supplementary Table I. *Scoping Reviews (PRISMA-ScR) Checklist*

| **SECTION** | **ITEM** | **PRISMA-ScR CHECKLIST ITEM** | **REPORTED ON PAGE #** |
| --- | --- | --- | --- |
| **TITLE** | | | |
| Title | 1 | Identify the report as a scoping review. | p. 1 |
| **ABSTRACT** | | | |
| Structured summary | 2 | Provide a structured summary that includes (as applicable): background, objectives, eligibility criteria, sources of evidence, charting methods, results, and conclusions that relate to the review questions and objectives. | p. 2 |
| **INTRODUCTION** | | | |
| Rationale | 3 | Describe the rationale for the review in the context of what is already known. Explain why the review questions/objectives lend themselves to a scoping review approach. | p. 4-5 |
| Objectives | 4 | Provide an explicit statement of the questions and objectives being addressed with reference to their key elements (e.g., population or participants, concepts, and context) or other relevant key elements used to conceptualize the review questions and/or objectives. | p. 5 |
| **METHODS** | | | |
| Protocol and registration | 5 | Indicate whether a review protocol exists; state if and where it can be accessed (e.g., a Web address); and if available, provide registration information, including the registration number. | N/A |
| Eligibility criteria | 6 | Specify characteristics of the sources of evidence used as eligibility criteria (e.g., years considered, language, and publication status), and provide a rationale. | p. 7  Table 2 |
| Information sources* | 7 | Describe all information sources in the search (e.g., databases with dates of coverage and contact with authors to identify additional sources), as well as the date the most recent search was executed. | p. 6-7 |
| Search | 8 | Present the full electronic search strategy for at least 1 database, including any limits used, such that it could be repeated. | p. 6-7  Table 1 |
| Selection of sources of evidence | 9 | State the process for selecting sources of evidence (i.e., screening and eligibility) included in the scoping review. | p. 7-8 |
| Data charting process | 10 | Describe the methods of charting data from the included sources of evidence (e.g., calibrated forms or forms that have been tested by the team before their use, and whether data charting was done independently or in duplicate) and any processes for obtaining and confirming data from investigators. | p. 8 |
| Data items | 11 | List and define all variables for which data were sought and any assumptions and simplifications made. | p. 8 |
| Critical appraisal of individual sources of evidence | 12 | If done, provide a rationale for conducting a critical appraisal of included sources of evidence; describe the methods used and how this information was used in any data synthesis (if appropriate). | N/A |
| Synthesis of results | 13 | Describe the methods of handling and summarizing the data that were charted. | p. 8  Table 3 |
| **RESULTS** | | | |
| Selection of sources of evidence | 14 | Give numbers of sources of evidence screened, assessed for eligibility, and included in the review, with reasons for exclusions at each stage, ideally using a flow diagram. | p. 7-8  Figure 1 |
| Characteristics of sources of evidence | 15 | For each source of evidence, present characteristics for which data were charted and provide the citations. | p. 8  Table 3 |
| Critical appraisal within sources of evidence | 16 | If done, present data on critical appraisal of included sources of evidence (see item 12). | N/A |
| Results of individual sources of evidence | 17 | For each included source of evidence, present the relevant data that were charted that relate to the review questions and objectives. | p. 9-17  Table 3 |
| Synthesis of results | 18 | Summarize and/or present the charting results as they relate to the review questions and objectives. | Figure 2-4  Supplementary Table II |
| **DISCUSSION** | | | |
| Summary of evidence | 19 | Summarize the main results (including an overview of concepts, themes, and types of evidence available), link to the review questions and objectives, and consider the relevance to key groups. | p. 17-21 |
| Limitations | 20 | Discuss the limitations of the scoping review process. | p. 21-22 |
| Conclusions | 21 | Provide a general interpretation of the results with respect to the review questions and objectives, as well as potential implications and/or next steps. | p. 22 |
| **FUNDING** | | | |
| Funding | 22 | Describe sources of funding for the included sources of evidence, as well as sources of funding for the scoping review. Describe the role of the funders of the scoping review. | N/A |

Supplementary Table II. *Articles that conceptually align to Public Health Perspectives (n = 8)*

| References  (Year, Country) | Title | Purpose | Key contexts of technology for aging | Implications for public health perspectives |
| --- | --- | --- | --- | --- |
| Cabrita et al. (2018)  Netherlands | Persuasive technology to support active and healthy aging: An exploration of past, present, and future | Multi-perspective analysis on technologies that can be used to support active and healthy aging in daily life | There are 4 domains of technology for aging from the perspective of inducing behavioral change, which is a key factor in healthy and active aging: 1) monitoring (measurement of daily behavior); 2) analysis and reasoning (deriving meaningful information through data analysis); 3) coaching (continuation of supportive behavior for individuals); 4) application (interface or tool that provides information to sustain desired behaviors). | Future older generations will already be familiar with using technology and can play an important role in system design. Cooperation among all stakeholders is needed, and if new smart devices based on large datasets are run anywhere at any time, then personalized medicine can be realized. Moreover, technology can support older people in playing a central role in the community. |
| Gandarillas et al. (2018)  Spain/Austria  /Slovenia | Merging current health care trends: Innovative perspective in aging care | Explore the best projects and implementation methods for innovating health care systems | Propose a home-based and community-centered integrated healthcare management system (IHMS); take an information and communication technology (ICT)-supported management and governance approach to improve health care delivery; and establish an individual data bank to use ICT for sharing. | Overcome the limits of existing e-health and establish health care e-governance to implement IHMS; develop an ICT/knowledge management (KM) tool that helps community-based, national, and international organizations to cooperate on information sharing, decision making, planning, and execution; simplify the structure of service provider agencies, which promotes inter-agency cooperation and allows for customized individual service, thereby improving cost-efficiency, prevention, chronic disease management, and QoL. |
| Koch (2010)  Sweden | Healthy aging supported by technology: a cross-disciplinary research challenge | Identify research gaps by viewing the role of technology for healthy aging from different scientific perspectives | Ambient assisted living is a comprehensive concept concerning how older people lead a safe and healthy lifestyle while maintaining their physical functions and social relationships in a familiar environment.  The roles of technology include the following, according to level of dependency in the aging process: (1) lifestyle management; (2) early disease detection; (3) assistance for daily life; and (4) disease management. | The most important task is to provide personalized service and meet the demands of the different stakeholders.  Social demand must be completely identified; legal and ethical safeguards must be thoroughly reviewed; and appropriate and utilizable technology solutions must be prepared.  Interdisciplinary research is required to obtain solutions by linking informatics and technology to each stage of aging. |
| Reeder et al.  (2013)  USA | Framing the evidence for health smart homes and home-based consumer health technologies as a public health intervention for independent aging | Systematic review of public health intervention using health smart homes (HSH) and home-based consumer health (HCH) technology | HSH/HCH technology is used to realize aging in place.  Three studies presented effective evidence, all of which used multifunctional technology, including activity sensing and reminders, while the evidence for population-based interventions was strengthened after 2005.  The review was limited to only 3 studies due to a communication gap between technology and health researchers, and many research findings were not reflected in daily life. | For the realization of aging in place at the population level, HSH/HCH research must be established according to evidence-based public health typology.  For such an intervention to be implemented within the community, reports on cost information and sustainable reimbursement models must be available.  Research is also needed on measures to enable the sound utilization of personal health records to develop a person-centered care model. |
| Riva et al.  (2014)  Italy | Positive technology for healthy living and active aging | Present how technology improves the quality of individual experience through a positive technology approach | Using technology to improve wellness and handling of the quality of experience with the goal of building the capacity to restore and maintain individuals, organizations, and society.  This positive technology approach was divided into 3 types:  Hedonic: technology for fun;  Eudaimonic: technology that allows for dynamic activity; and  Social/Interpersonal: technology that strengthens communication links. | Technology can be used to resolve the increasing disease burden and costs due to the growing older population.  Healthcare organizations can encourage older adults to play a more active role in their own health and wellbeing, while positive technology can present methods for enhancing the quality of individual experience.  The approach can be expanded and applied to any sector in which healthcare services are provided. |
| Satariano et al.  (2014)  USA | Aging, place, and technology: toward improving access and wellness in older populations | Introduce technology for the realization of aging in place and proposed measures for increasing accessibility to technology among older people | Realization of aging in place requires an increased level of evidence by testing technology among various older people. New collaborations and partnerships between multidisciplinary teams, the elderly, and caregivers are needed.  Innovatively secure funding and increase the safety of technology use by older people. | The use of ecological models is increasing in the public health sector, which indicates aging in place.  Accordingly, technology can enhance aging in place by increasing the adaptability between individuals and the environment, while reducing public costs.  Strategies are required for organizing, funding, disseminating, and sustaining technology. |
| Turchetti et al.  (2011)  Italy | Technology and innovative services | Introduce technology that can take a problem-solving and service approach applicable to the aging population | Proposed services science, management, and engineering (SSME) for the third age and health care. It is necessary to solve the following two problems:  Innovative high-tech products must be included in long-term care and accident services.  A multidisciplinary team comprising management, biomedical engineers, and clinicians must be assembled to continuously upgrade products and services. | Such a methodology can promote contributions and partnerships between the insurance sector and industry-producing technology for the older generation.  Since increased social costs are expected, it will be an appropriate solution with high acceptability and a relatively lower cost.  Using long-term care as an example, technology can be used for short term control of the environment. Additionally, commercial robots can be introduced to help with household chores in the intermediate period. Further, third generation eCare services that assist cognitive function and remote monitoring can be provided. |
| van Bronswijk et al. (2009)  Netherlands  /USA | Defining gerontechnology for R&D purposes | Identify the field of gerontechnology and present its characteristics | Gerontechnology has the ultimate goal of facilitating successful aging by enhancing older people’s quality of life, and it encompasses all technologies through the entire life course. To accomplish this, it is necessary to consider changes at the environmental and individual levels, as well as develop an interdisciplinary approach between experts in the fields of technology and gerontology. | Gerontechnology is an important means of assuring sustainable development and social sustainability.  The goal of gerontechnology in the public health sector is to integrate the application of science with policy to reduce inequality by providing the best possible health to as many people as possible. |
